# Swedish managers’ experience-based understanding of the Capacity to work in employees with Common Mental Disorders: a Focus Group Study

**DOI:** 10.1007/s10926-022-10029-8

**Published:** 2022-03-04

**Authors:** Ellinor Tengelin, Gunnel Hensing, Kristina Holmgren, Christian Ståhl, Monica Bertilsson

**Affiliations:** 1 Department for Health Sciences, University West, SE-461 86 Trollhattan, Sweden; 2 Insurance Medicine, School of Public Health and Community Medicine, Institute of Medicine, University of Gothenburg, SE-405 30 Gothenburg, Sweden; 3 Department of Health and Rehabilitation, Institute of Neuroscience and Physiology, The Sahlgrenska Academy at the University of Gothenburg, SE-405 30 Gothenburg, Sweden; 4 Department of Behavioural Sciences and Learning, Division of Education and Sociology, Linköping University, SE-581 83 Linköping, Sweden

**Keywords:** Focus groups, Occupational health, Psychiatry, Qualitative research, Work performance

## Abstract

**Purpose:**

Understanding of the capacity to work among employees with common mental disorders (CMDs) is important, but contemporary knowledge on this issue lacks the managers’ perspective. The aim of this study was to explore and describe managers’ experience-based understanding of capacity to work in employees with CMD.

**Methods:**

A qualitative focus group study was designed. Managers with experience in supporting employees with CMD were recruited via organizations and networks. Eight focus group interviews with 31 participants took place.

**Results:**

The analysis resulted in five categories. (1) Capacity to mentally focus on work tasks decreases or disappears, with negative consequences for work output. (2) Capacity to commit to continuous and coherent task changes, making tasks that span longer periods of time difficult. (3) Capacity to independently adapt to the needs of the situation decreases, and employees need more guidance and instructions than usual. (4) Capacity to keep up professional appearances is reduced, and the employees struggle with the professional role. (5) Ability to interact socially and professionally decreases, which potentially causes conflicts at the workplace.

**Conclusions:**

This study adds managers’ perspective to the increasing knowledge on how capacity to work is influenced by CMDs. Managers understand CMDs in employees as changed, reducing the capacities needed for occupational functioning. A deeper understanding of reduced capacity to work is needed to adapt workplaces, and our findings can facilitate work accommodations for employees with CMDs.

## Introduction

Understanding of the capacity to work among employees with common mental disorders (CMDs) is important, but contemporary knowledge on this issue lacks the managers’ perspective. Work capacity is a prerequisite for being part of a working life and being affected by CMDs has significant implications for individuals and their workplaces. Working life is an arena with changing and dynamic conditions, and capacity to work can be understood as the product of the requirements and expectations in this arena [1]. Managers have an overview of these factors and the conditions that shape the capacity to work at their workplaces; they are familiar with their employees’ capacities as managers are responsible for the work environment and for tailoring accommodations, preventing sickness absence, and supporting return-to-work after sickness absence [2, 3]. However, although employees’ own experiences of CMD-related work capacity have been explored previously [4–7], managers’ perspectives are less well known, even though their perspective can contribute useful knowledge.

Since self-assessed work capacity among newly sick-listed employees with CMD predicts return-to-work [8–10] as well as future work participation 1 year after baseline assessment [11], CMD-related work capacity needs to be explored from different perspectives. However, few studies have focused on work capacity in individuals while at work, and, as described above, managers are an underutilized resource in this matter. Rationales for exploring work capacity include the increased risk of negative work outcomes and both short-term and long-term sickness absence and high societal costs [12, 13]. CMDs are prevalent in the Swedish workforce [14, 15] and internationally. The Organisation for Economic Co-operation and Development (OECD) has reported that 15% of the working age population are affected by CMDs and 72% of those reported decreased work capacity, compared with 25% of their peers with somatic disorders [13]. Both OECD and authorities in Sweden conclude that we need knowledge about CMDs’ impact on work capacity [13, 14]. Managers’ understanding of how employees’ capacity to work is affected will provide a new angle to better understand such impact. More thorough information about employees’ capacity to work would enable managers to better support employees, but also help them with early identification of employees with CMDs and to better tailor work accommodations and facilitate back-to-work rehabilitation after sick leave [16, 17].

CMDs include depressive and anxiety disorders [18]. Symptoms range from mild to severe and can last from months to years. Of the main symptoms, fatigue and concentration problems are common; they are associated with slow treatment response [19] and reported by employees to affect capacity to work [4]. Exploring the capacity to work means attending to situational and contextual aspects of work, such as the type of job and the conditions in the workplace [20]. The same mental symptoms can have different consequences for the capacity to work in different individuals, and therefore the phenomenon itself needs to be explored, rather than equating it with symptoms or impairments of mental ill-health.

Theoretically, the capacity to work construct can be described by the conceptual framework of individual work performance (IWP), which builds on four domains of performance at work and adds the employer perspective [21]. *Task performance* includes the proficiency with which employees perform central job tasks. *Contextual performance* includes actions that support the organizational, social, and psychological environment in which job tasks are carried out. *Adaptive performance* refers to the employee’s ability to adapt to changes in a work system, and *counteractive behaviour* includes behaviour that harms the well-being of the organization; for example, sickness presenteeism and sick leave. Employers have an overview of all four domains, which highlights the importance of exploring capacity to work from an employer perspective. However, while theoretical frameworks such as IWP illustrate the particulars of general work capacity, they need to be complemented with theoretical knowledge of how each domain can be affected in specific disorders, thus helping managers and workplaces deal with and support employees with CMDs.

As far as we know, only one study has explored work capacity from the managers’ perspective [22], including managers’ experiences of employees’ mental health problems in general, not particularly CMDs. Because psychiatric disorders have a unique set of symptoms and impairments, the results of such a study have limited transferability to particular disorders, such as CMDs. Other qualitative studies have described some findings on managers’ experiences of employees’ capacity to work, even though their main purpose was not to explore that phenomenon [16, 23, 24].

There are several reasons to explore the phenomenon of capacity to work in people with CMDs from the managers’ perspective. First, most studies explore work capacity from the perspective of the individual, and it is often argued that people with mental ill-health tend to undervalue their capacities which might bias study results [25]. When physicians assess capacity to work and the need for sickness absence, findings show that they request what they see as more objective evaluations of the patients’ capacity, for example from the patient’s manager, because they are considered by the physicians as more valid and trustworthy than the patient’s own statements [26]. Also, among other stakeholders in return-to-work processes managers have been identified as key persons in relation to employees’ work capacity [3]. Second, managers have an overview of the workplace, including the interaction among co-employees and the work output, for example, client satisfaction and ability to keep deadlines. Thereby, it can be expected that managers’ experiences can add to the knowledge base about capacity to work and be used to support employees in return to and remaining in work. Third, managers find it hard to understand capacity to work among employees with CMDs, and experienced managers’ understanding of the phenomenon can be used to support their less knowledgeable likes [16, 17].

### Aim

The aim of this study was to explore and describe managers’ experience-based understanding of capacity to work in employees with CMDs. Through their experiences of encounters with and support of employees with CMDs, managers can be expected to have an overall understanding of their employees’ capacity to work.

## Method

### Study Design, Participants, and Procedure

This study is part of the New Ways – Mental Health at Work programme and the Managers’ perspective – a missing piece project. The study was designed as a qualitative focus group study, chosen because of the interactive and explorative character of data collection [27, 28]. Focus group discussions should result in more than the sum of separate interviews, because participants both query and explain themselves to each other [27]. The focus group setting offered the participants an opportunity to identify and discuss their experiences of capacity to work among employees with CMDs.

Inclusion criteria for the study were (1) being manager and (2) having professional experience of supporting an employee with CMDs. To recruit participants, we approached human resources departments in public and private organizations (e.g. municipalities, hospitals, universities, large companies) and employer networks, informed them about the study and asked for their interest and the possibility of passing on the study invitation to their first-line managers. Managers interested in participating sent an application of interest to the last author, who contacted each person and checked that they fulfilment the inclusion criteria (excluded *n* = 8). Thereafter, 41 managers received information about the study, the focus group method, and the themes to be discussed during the interview. They were invited to one of eight focus group sessions; 10 managers were not being available on the set date, cancelled, did not show up, or withdrew. Because we recruited at an organizational level, all but two focus groups were composed of managers from a single organization. Two focus groups consisted of managers from small enterprises. Thus, in six focus groups, the participants knew each other and shared the same context for their managerial work, whereas the participants in the remaining two groups did not. Four of the sessions included five or six participants, three sessions included three participants and one session included two participants.

At the beginning of each focus group session, the participants signed a document ensuring their informed consent and filled in a form on demographic information (Table 1). They were encouraged not to name individuals they were referring to or give out any information that could lead to the identification of any one employee. No incentives were offered other than reimbursement for travel expenses. The study was approved by the Regional Ethical Review Board in Gothenburg, Sweden (Dnr 165-17).

**Table 1:** Main interview questions

Opening questions	What are your experiences of subordinates with depression or anxiety?
	How do you notice a subordinate has this problem?
Key questions about capacity to work	How do you perceive that the capacity to work is affected in subordinates with depression or anxiety? Give concrete examples
	Is there anything that characterizes these people when it comes to capacity to work?
	In what way is the depression or anxiety itself affecting their capacity to work?
	In what way is the work environment affecting their capacity to work?
	In what way are the work tasks affecting their capacity to work?

### Data Collection

The interview guide was piloted in a test focus group and worked well; no changes were made (Table 2).

**Table 2:** Demographics of the study participants

Characteristics	Number
Sex	
Women	21
Male	10
Age (years)	
Range	28–62 years
Mean	49 years
Level of education	
Upper secondary school	5
Degree from college/university^a^	23
Other post-secondary education	3
Sector	
Private	14
Public	17
Industry^b,c^	
Blue collar	7
White collar	11
Pink collar	15
Managerial position	
Senior manager	1
Middle management	7
Middle management/first-line manager	23
Years of managerial experience^d^	
Less than 5 year	8
5–10 year	6
More than 10 year	16
Experience of subordinates with depression and/or anxiety disorders	
In the last 2 years	
None	1^e^
1–2 subordinates	13
3–5 subordinates	15
More than 5 subordinates	2
More than 2 years ago	
1–2 subordinates	7
3–5 subordinates	11
More than 5 subordinates	4
None/do not know	9

^a^Minimum 3 years

^b^Blue collar, working with things; white collar, working with symbols; pink collar, working with people

^c^Adds up to 33 (2 participants were categorized as working in both white collar (research) and pink collar (education) industries)

^d^Missing information *n* = 1

^e^Had experience of subordinates more than 2 years ago

Eight focus groups were carried out between November 2018 and March 2019 with a total of 31 participants from the public or private sector.

The interviews were moderated by the last author (MB) who has extensive experience in moderating focus groups on CMD at work. Three experienced co-moderators participated, KH (*n* = 2) or CS (*n* = 3), and in one session another researcher. They assisted in keeping the discussions lively, interactive, and relevant to the study aim. In two sessions, only MB moderated because there were only a few participants. Probes were used to facilitate and deepen the discussions. The discussions were audio-taped, transcribed verbatim by a professional transcribing firm and the transcripts were checked for accuracy by MB.

### Data Analysis

Qualitative, manifest content analysis [29] with an inductive approach was used to analyse the data; the first author was the main analyst. All co-authors (experienced in qualitative analysis, mental health, sickness absence and work capacity research) took part in identifying content relevant to the study aim, either through reading the transcripts or listening to the audio files. This joint effort created a wider analytic space than is possible for just one author [30]. From the selected content, the first author identified meaning units, gave each unit a code, and grouped and re-grouped the codes into sub-categories and categories by comparing the similarities and differences. In the final categories, codes that share some manifest content relevant to the study aim were grouped together. Relevant passages in the transcripts were re-read regularly to avoid losing the sense of the context from which the units were taken. The author group discussed the preliminary analyses throughout the analytic process. NVivo software version 12 [31] was used in the process of sorting, arranging, and labelling the data.

## Results

The findings describe the managers’ experience-based understanding of employees’ capacity to work with CMDs. The managers understood employees’ decreased capacity to work in the sense of a changed capacity; employees with CMDs gradually changed from their usual way of handling work tasks efficiently to partial or complete incapacity. The managers learned about this changed capacity through conversations with the employees, colleagues, clients, and customers, and by observing the employee at work. CMDs changed and reduced the central capacities that were needed to carry out work tasks and assignments satisfactorily, presented as categories in the following sections. Abbreviations are used for informant (Inf.) and moderator (M.).

### The Capacity to Mentally Focus on Work Tasks Decreases or Disappears

The managers described an essential capacity, which in their understanding decreased or disappeared with CMDs: being mentally focused, present, attentive, and concentrating on the routines and tasks at hand. Few tasks could be completed without some level of focus and mental presence, and the managers described that even the most basic routines of work can be forgotten under the influence of CMDs, such as clocking in at the start of the day. This seemed to happen because the employees were preoccupied with their personal thoughts and troubles, and therefore less able to engage in the work situation and its demands. They appeared to be in their own world, unable to keep up at the usual pace. The managers described how an inability to concentrate affected the performance of both complex intellectual tasks and manual tasks. For example, in data programming work, employees were required to maintain a continuous dialogue with the customer and try different solutions; there were no right answers or instructions to follow. A sharp mental focus is needed to succeed at this. In assembly work, focus is needed to keep up the speed on the line, making it impossible to let one’s mind wander even for 10 s. Decreased capacity to focus could be even more impaired due to difficulties in shutting out disturbing events and surrounding noises.

In the managers’ experience, CMDs affected memory, and employees with CMDs forgot things they had promised to do. The managers experienced that work tasks were not finished, sometimes not even started. It seemed to them that their employees “lose themselves along the way”. It was difficult for the employees to concentrate on one task until finished; alternating between different tasks during the workday was also difficult. The managers described how strenuous it was for employees to change their focus on the work: they may be able to cope with doing one specific task, but when something else required attention, it became too much. They struggled with keeping track of the chronological order of carrying out work assignments and tasks, which resulted in a messy situation in which no part of the task got done properly.

The managers also illustrated the lack of mental focus by a decreased capacity to receive and communicate information. They felt that the employees’ focus was “clearly elsewhere”. Employees within home care services may not accurately read the labels of medicines before they give it to patients, causing the managers to worry about patient safety. In their experience, it “just doesn’t work” for an employee to read a text, digest it, and then convey the content to another person. The ability to follow a line of reasoning from beginning to end in a given situation appeared to be reduced. CMDs hindered the ability to think analytically and logically, and to take in and process sensory information.

Inf. 1 (female, white collar): If they do their thing, they don’t really get to the detail [of the work task] because they don’t have the ability to deepen their thoughts, sort of, they reach a stop. It is as if everything is limited for them, viewing and hearing and thinking ability… especially this capability of getting into the details of something, thinking a thought to the fullest and analysing it; sort of, it is cut off.

Inf. 2 (male, white collar): Problem-solving is really difficult, right? When something doesn’t work the way it’s supposed to, what is it that is wrong.

Inf. 1: Yeah, and that creates stress in itself which makes it work even worse. (FG5)

### The Capacity to Commit to Continuous and Coherent Tasks Changes

In the managers’ understanding, tunnel vision seemed to reduce the ability to get a coherent overview, time frame, and context to fully carrying out tasks. This caused problems in situations that required attention to a complete time span. Even though daily tasks may work well, fragmented assignments that required planning, analysis, and responsibility for events over time were hard to carry out. Tasks that were concrete enough to check off a list and did not require any more reflection once they were done work better and are often preferred.

Inf. 1 (male, pink collar): There are different events during the year and at certain times they do certain work tasks….

M.: Other than the regular ones?

Inf. 1: Like in school… I can name this one example; in school, we have parent-teacher conferences, once per term… In things that happen only once per term, you [as a manager] discover that they cannot seem to handle it, these things that go beyond the usual work.

M.: The usual teaching.

Inf 1: Yeah, […] there are work tasks that they do maybe once or twice per year, and other things are just ongoing… certain things are ongoing and then this bigger thing comes, that requires them to handle more contacts, a bit differently… that is one of those things.

Inf. 2 (female, pink collar): And I also think that the employees who I’ve had who have problems with depression and anxiety, they make the daily tasks work overall. But when it comes to, as you say, certain things, the tasks become too big, too difficult, too complicated. That is my experience too. (FG1)

Work activities that were fragmented during the day were harder to grasp for employees with CMDs. As a result of their tunnel vision, they struggled to connect them. CMDs interfered with the capacity to “tie up loose ends”, which included the ability to keep things in mind during the entirety of a workday and remembering the purpose of decisions and actions. Problematic tasks could be following someone’s development over time, such as the grading of students at the end of a school year, which presumed that the employee remembered and accumulated knowledge and experience over time. Another example was the difference between the basic nursing of children compared with the long-term engagement of children’s development. Work tasks that were never completely finished, such as continuous developmental work, were difficult. In the managers’ understanding, these kinds of work demands could “push the employee over the edge” and force them into sick leave.

### The Capacity to Independently Adapt to the Needs of the Work Situation Decreases

CMDs complicated the manageability of demands of working independently and being flexible in adapting to the changing needs of the work situation. The managers expected employees to handle their work role on their own, and if need be, step out of their comfort zone. These capacities may fail under the influence of CMDs, described below in two sub-categories.

#### Difficulties in Working Independently

The managers counted on their employees’ ability to take responsibility for their part of work and carry out their tasks independently, but they saw that this capacity was difficult to uphold with CMDs. Instead, employees depended on close guidance in their work, and the need for validation was extensive. The expectation that they can work independently could itself trigger more anxiety. The managers described how CMDs made the employees stop believing in their competence and that they seemed “incapable of making their own decisions” (FG5). Instead, they asked colleagues and managers for detailed instructions, so that the execution of the task could not go wrong. They checked whether they understood the instructions correctly several times and appeared to wrestle with a fear of making mistakes. Difficulties may arise for example, in client meetings, where employees were supposed to run the dialogue independently and pose the right kinds of questions to get the necessary information to handle the client’s needs. Sticking to routines that can be done almost automatically, provided feelings of security.

[Employees] have to be able to work independently and there are limits for how much you [as a manager] can be expected to clarify and describe the work tasks. And if they have a strong need to get this validation of what is really the right answer… there is a limitation in the way we work. They have to work independently from a job description that is often rather abstract. And this is something this group [of employees with CMDs] seems to have a problem with, sometimes. To understand… what am I supposed to do, what is it that isn’t working, why isn’t it working… (FG4, male, white collar).

#### Difficulties in Leaving One’s Comfort Zone

The managers described how employees with CMDs depended on predictability in their workdays. These employees had a difficult time acting on changed and unexpected conditions in the work situation and found it difficult to adapt quickly to these. Instead, managers saw them having a fundamental need for predictability in their work environment. The need to know in detail what was going to happen in a certain situation increased with CMDs, and it was difficult for the employees to handle situations where they had to think anew or creatively. It was exhausting for employees not knowing in advance which colleague or client they were supposed to work with, ”they don’t really manage to rethink” (FG1) the situation if plans for their work shift were changed.

In the managers’ view, CMDs caused difficulties with elements that deviated from the everyday workplace routine. At some workplaces, business trips or meetings outside the office were not part of the daily tasks. When they occurred, the employees must leave their comfort zone to meet the needs of these specific situations. With CMDs, this was strenuous, and made the employees react with stress and irrational behaviour, such as cancelling these meetings at short notice.

There is always a cancellation, just before they are supposed to go, when it comes to conferences and things like that. They lock themselves into the safety of their own space where they have control over each element, where it’s always the same. “This is what my day looks like and what every day should look like” and when it comes to non-standard work elements it gets… it influences their work a lot. It can be anything from going to a seminar somewhere to mandatory conferences where they are supposed to sketch out a strategic direction for the whole group, or a team-building session, or things like that. (FG7, female, white collar)

### The Capacity to Keep Up Professional Appearances is Reduced

The managers found that employees’ capacity to keep up appearances and maintain their professional role during work is impaired. They had a hard time containing their emotions, even for apparent bagatelles, sometimes resulting in mental and physical breakdowns and sudden outbursts. In the managers’ understanding, CMD weakened the “shield” that made it possible for employees to keep their feelings to themselves. This was problematic because employees were expected to avoid too much of their emotions and vulnerability during their work. To get work done, employees struggled to hide their emotional state, and sooner or later the managers observed that something was wrong.

In the managers’ view, the ability to keep up professional appearances could be especially problematic in professions in which the employees were exposed to scrutiny from others. One example was cleaning jobs during office hours, where the cleaners are expected to change between blending into the environment and to socialize. Their troubled feelings must not be shown, since “you cannot be a disturbing element when you clean” (FG3). This balance became difficult to uphold, which made people around the employee worry about the employee’s health. To work as a teacher required the employee to stand alone in front of a whole class, answer questions and handle criticism and external evaluations. Dealing with relatives was part of many professions and required the employee to handle being questioned, as well as to explain the motivation for decisions. CMDs challenged them to keep up appearances and keep track of their own emotions. The managers described how the employee instead “contaminated” clients, pupils, and work environment with their state of mind.

It is fairly obvious, when someone isn’t feeling well, in a small group like this; it is almost like a family and working in teams. It affects the whole group in many ways and most clearly […] it was outbursts and strange actions towards their colleagues that spread a weird atmosphere at the company, escalating over time. In one case, there was a lot of acting out. Then, we had another case where they locked themselves in their office; they came to work, closed their door, and didn’t come out until it was time to go home. […] we work really hard at transparency at our company, we work together, in teams… (FG7, female, white collar).

### The Capacity to Interact Socially and Professionally Decreases

The managers noticed that relationships in the workgroup around the sick employee got complicated, and the mutual interaction process between the employee and other people, be it colleagues, clients, customers, patients, or their relatives, became challenging for the employees to handle. The managers’ understanding of the influence of CMDs on the mutual interaction process between the employee and other people is described in three sub-categories.

#### Difficulties in Interacting with Colleagues

The managers described how the changed work behaviour due to CMDs affected collegial situations. The social interaction in workgroups may become tense, unbalanced, and even dysfunctional around the employee. The employees may come to work, but withdraw from social gatherings, keep to themselves, and hide behind (sometimes symbolic) walls. In some professions, it was acceptable to work from home, enabling the employee to totally cut themselves off from social relations with others at the workplace. Managers described it as a tendency to “lock themselves inside a box” to avoid social interaction (FG4). The managers also observed how professional communication around the employee became difficult, as co-workers avoided bringing up critical things to discussion, such as the quality of work output. Both coworkers and managers made an effort to carefully choose their words when communicating with the employee because of the misunderstandings that could easily arise.

Inf: I try not to talk too much about… I try to be very frank about what I think, because I can see that the others walk on eggshells [around the employee with CMD] and that they don’t dare to ask questions… they avoid it, [but] you have to be as frank as possible, even though it is hard.

M: With the employee?

Inf: Yes, with the employee, […] the colleagues also need to be frank. (FG2, female, pink collar)

Tasks that required communication and collaboration could get impaired when the employees “take their own path” (FG5). Collaboration required that colleagues communicated around shared thoughts and work processes, and according to the managers, this was something that employees with CMDs seemed to avoid. This affected the output of work because work assignments were not considered from the perspective of the whole group. Employees with CMDs focused on their own part instead. Assignments tended to be turned in late and did not meet the expected standards.

#### Difficulties in Interacting with Clients, Costumers, Patients, and Others

The managers observed how employees with CMDs lacked the tolerance that was necessary in interactions with, for example, pupils, children, people with psychiatric diagnoses, the elderly and their relatives, and that they could get defensive in communication with them. The employees had a hard time picking up and acting on the signals from other people. In a situation where it was obvious that a child needed attention, an employee with CMDs could avoid the necessary interaction and instead start doing some practical tasks that did not need immediate consideration. In many human service contexts, it was essential to be able to read social situations and anticipate what would happen next and what the needs of the group were. The managers described this as a certain capacity with high demands on attentiveness – to be “one step ahead”.

Inf. 1 (female, pink collar): They often avoid interaction with the youths. They do other things… unload the dishwasher; they don’t see that there are youths who… they remove themselves from a professional interaction. If someone is acting badly, it is their colleagues who will have to take care of that.

Inf. 2 (female, pink collar): […] they can sit there hour after hour [with a client] even though it might not be necessary. It is not necessary for the client, but it is necessary for them because they feel they are doing a work task, and they can manage it for the two hours they are supposed to be at work. (FG2)

#### Difficulties in Interacting with the Manager

The managers experienced that employees with CMDs started to shy away from interaction instead of discussing problems directly with the manager. Alternatively, they started to approach the manager with criticism and sometimes discontent about the working conditions. In the managers’ interaction with employees, their choice of wording became more important when the employee had CMD, and the managers felt they may have to watch what they said. This could make communication complicated if the employee focused on other words, rather than the meaning of a conversation. The managers also had to put effort into convincing the employees that they were good enough by telling them “you’re good, you’ve got this, you do have the qualifications” (FG4).

Instead of the normal employee-manager interaction, the employee with CMD could start confiding in the manager. The professional boundary may be crossed in these conversations. The employee tended to make sure they got time alone with their manager, and when the manager visited the workplace, the employee could be eager to get the manager’s attention. Overall, this manner of interaction changed their relationship into something the managers perceived as a “therapist role”.

This is my experience of those [employees with CMDs] who I have met; it feels like you’re walking on ice, sometimes. Sometimes, certain conversations can work just fine, but then they can get stuck on one specific word, and then, we have to discuss that word next time. And I am not trained for that… I am not a psychologist, but I have to… (FG2, female, pink collar).

## Discussion

This study has explored and conceptualized capacity to work in employees with CMDs from the experience-based understanding of managers, highlighting how managers understand limitations on the capacities needed for work related to CMDs. The analysis resulted in five categories: the capacity to be mentally focused on work tasks is essential but lost; work tasks fragmented in time or context are severely problematic to pursue; working independently in a changing situation fails; keeping up professional appearances and roles is hard; and processes of social interaction are changed. The findings are the result of the inductive content analysis of managers’ experiences, and it cannot be expected that all employees with CMDs experience all constituents displayed. Capacity to work is a dynamic relation between the employees’ capacity, the characteristics of the work environment and the demands of the work tasks, which differs between employees [1].

Managers’ perspective is one of many that can shed light on capacity to work while affected by CMDs. With their overview of the workplace, focus on work output, and knowledge about how CMDs can be expressed in different individuals, managers understand capacity to work in employees differently than the employees themselves [3, 26]. However, one also needs to be aware of the problematic situation for managers, as many employees choose not to disclose their CMDs and decreased work capacity to their managers [32]. A study conducted in Sweden reports that about 60% of managers of employees with CMDs have been informed by the employee him or herself [33]. Both managers and employees struggle with approaching the situation [32, 34] and our findings could aid managers to be aware of signs of decreased capacity to work and to identify employees with greater need for early support and work accommodations.

Our findings correspond well with the IWP framework [21], showing the “mismatch” between managers’ expectations on individual work performance and how they understand employees’ capacity to fulfil these expectations under the influence of CMDs. The first two categories on mentally focusing and committing to work tasks concern *task performance* in the IWP, referring to the competency with which one performs work tasks. Competency was reduced in such a way that the changes in work output were noticeable to managers, as expressed in descriptions of decreased focus, memory, and problem-solving skills. The influence of CMDs on work performance that could be classified as task oriented has been suggested in a previous interview study with managers [24], who said they observed changes in work output among employees with CMDs similar to the changes the managers described in our study. These changes included less satisfactory performance of work duties, fluctuations in productivity, failure to complete assigned work or complete it in the required manner, and deterioration in the quality of work. Some of our findings on task performance also show similarities with a study investigating managers’ perceptions of the impact of mental health problems on work ability [35]. Even though that study concerned a wider perspective on mental ill-health than CMD entails, it shares some results with our current study, such as reduced ability to focus [35].

The third category concerned employees’ reduced ability to adapt to the needs of the work context and corresponds to the domain of *adaptive performance*. Adaptive performance includes generating new, innovative ideas, adjusting goals and plans to the situation, and being open-minded and understanding towards others [21]. This performance domain also includes aspects that were found in our fourth category on reduced ability to keep up professional appearances, such as problems with remaining calm in professional situations, acting appropriately at the workplace, and not losing too much of the expected professional facade. Managers have also observed lowered tolerance to the demands of the work environment among employees with CMDs [24], which can be seen as a reduction in the adaptive performance. Managers have identified difficulties adapting to changes in a group of employees with mental health problems [35].

The fifth category concerned employees’ actions in the psychosocial context at work. The capacity to engage in processes where it is essential to listen, cooperate, and adapt to another party was affected, as understood by managers. This category has some overlap with the *contextual performance* domain of the IWP framework, including behaviours going beyond formally prescribed work goals; for example, showing initiative, enthusiasm, and commitment. In a previous study, managers described that difficulties in interaction and collaboration with colleagues, costumers, and managers occurred in this group of employees; for example, they became hostile [24]. Communicative and collaborative capacity is essential for the workplace to function and when these competencies are impaired, employees struggled with the processes that require social interaction, such as professional communication with customers and keeping a professional distance from members of the workgroup. To our knowledge, the specific communication between managers and employees is not described in depth in earlier studies. Our findings show that managers’ understanding of the changed communication between the manager and the employee with CMD as something that can be complicated to handle, adding to the image of CMDs as severely affecting contextual performance at the workplace.

*Counterproductive work behaviour* is a domain of the IWP framework that cut across several of our categories. It entails different behaviours that might harm the well-being of the organization [21]. In another study [16], managers’ saw that employees with CMD expressed increased sensitivity that influenced work performance. Employees were described as snapping or bursting into tears in social interaction, behaving inappropriately towards managers, colleagues, and customers, and losing focus on work tasks. In a larger group of employees with mental health problems, managers observed increased sensitivity and frustration in employees with mental health problems when interactions at the workplace did not work as they used to [35].

Many of the changes in employees caused by CMDs have previously been described by employees themselves. Employees find it challenging to concentrate, keep up with the speed of the work, and take initiatives, as well as mentally focusing on the work tasks and not postponing things that must be done [4, 6, 7], which illustrate task performance aspects. Other reduced capacities experienced by employees include aspects of the contextual work performance. Interpersonal tasks are especially challenging to handle because it can be difficult to listen to others, collaborate with colleagues, express empathy and anticipate other people’s needs [4, 6, 7]. Reduced adaptive performance can be seen with regard to employees’ difficulties in handling deviations from the everyday routines, new tasks or settings [36]. In this area of performance, we noted a difference between employees’ descriptions in a previous study and the findings in this study. Although employees themselves experienced that they have to put up a professional facade in order to get through the work [36], the managers observed that the employees did not succeed in this; they understood the failure to keep up a professional facade as a reduced adaptive performance. Further, employees with CMDs have described that order in the workplace order gets disrupted due to their decreased capacity to work [36]. They experienced that they had to burden their colleagues by asking for extra help, having to rest in the middle of the day, forgetting details about the work tasks, misunderstanding and overreacting, and not being able to carry out work if the everyday routine was not followed. These kinds of disruptions illustrate counterproductive work behaviour.

Employees find it difficult to grasp the situation when having CMDs [7], which underlines the managers’ important supportive role for this group [2]. However, shortcomings have been identified in managers’ work accommodation for employees’ returning to work following CMDs. A study identified that accommodations directly related to work aspects were common (e.g. modifications of tasks and schedules), but fewer accommodations were related to the social environment or the employees themselves [37]. Managers have been shown to accommodate work to a lesser extent for employees with CMDs than for employees with physical disorders [38], despite employees with CMDs reporting a greater impairment in capacity to work [39]. The reason for this could be that managers do not have sufficient knowledge of how capacity to work is influenced with regard to CMDs. Our study can help managers in this matter since the findings are based on managers’ perspectives and on experienced managers’ own descriptions.

It is easy to focus only on one side of individuals’ decreased capacities, which was what the managers did in the interviews, partly because of the design of the interview questions (Table 2). However, having CMDs does not have to mean that employees cannot fulfil their tasks. Participants with CMDs in an earlier qualitative study were all working, although a few of them only part time [4]. Furthermore, as discussed above, the findings are to be interpreted as a range of possible incapacities. However, there are indications that employees with CMDs may use all their energy on successfully maintaining their capacities at the workplace, showing no reduced work capacity, but ending up with no energy to do things in their free time [4, 26, 36].

### Methodological Considerations

The focus group discussions provided the managers with opportunities to discuss their own and shared understandings. In six of the groups the participants came from the same organization, whereas two groups included participants from different organizations. This may have influenced the social climate in the groups, because members from the same organization already have established their relationships, potentially impeding the discussion. However, in all eight groups, the participants shared their experience of managing one or more employees with CMDs, and this kind of homogeneity is considered important to create group identity and to encourage interactive discussions [40].

Our findings are based on experiences from informants with an overview of employees’ actions and behaviour at work, showing overlap with findings that have been described previously from the perspective of the affected employees [4, 6, 7]. Studying the same phenomena from different types of informants can be seen as a form of triangulation [41], strengthening the credibility of both the present and previous findings. The trustworthiness of the findings is strengthened by the reflexive analysis procedure and lengthy citations from the data. A researcher not previously involved in the research group’s studies (ET) performed the main analysis, while continuously discussing with the co-authors, who have different areas of expertise within the field of CMDs and capacity to work.

In the data collection process, no distinction was made between various kinds of CMDs, because they share similar symptoms and impairments [42]. The diagnosis does not determine an individual’s capacity to work [43], and this can also vary between individuals with the same diagnosis. Further, it could not be assumed that the participants would have sufficient knowledge about the diagnostic system to make the distinction.

The selection of participants is vital in understanding our findings and how they may be transferrable to other contexts. The participants in our study were mostly female and well-educated (Table 1). A recent study in Sweden showed that male managers were more likely to report negative attitudes to depression than female managers, which could be one reason for fewer male participants [44]. Although managers from mainly male-dominated sectors participated, a male-dominated focus group of participants could have expressed other understandings of the phenomena.

The decreasing capacities among employees, experienced by the managers in this study, are not the same as the actual capacities of these employees. This study set out to explore managers’ understanding, as this is a field that is underexplored. However, the managers’ understanding can be based on preconceptions or individual biases. In order to avoid such preconceptions, the inclusion criterion was to have professional experience of supporting an employee with CMDs. In the focus groups, the participants were encouraged to give detailed descriptions of the work capacity of these employees. Furthermore, due to the fact that disclosure is a main problem for employees with CMDs, it might be that the managers’ experiences are based on more severe cases of CMDs or on employees in jobs where it is more difficult to hide their problems.

### Implications for Research and Practice

The present study adds a complementary angle to earlier qualitative studies on capacity to work [4, 6, 7]. Future qualitative studies could contribute experiences from managers in other workplaces and contexts. To further understanding of CMDs and their impact on capacity to work, epidemiological studies in both clinical and working populations should be performed. However, that is currently problematic, because of the lack of instruments that measure the particularities of work capacity in people with CMDs [45, 46] as well as the lack of instruments that take a comprehensive biopsychosocial approach to the individual with CMD [47].

Our study can be used to shine a light on managers’ prerequisites for fulfilling the obligation of work accommodation and how employers and managers can aid in preventing these disorders from deteriorating. In order to support employees with CMDs or accommodate work for them, managers need to understand how work capacity is affected. More in-depth knowledge of the capacity to work from a workplace perspective can be used to expand managers’ knowledge and competence regarding maintaining employees’ capacity to work and tailoring work accommodations, which might prevent sickness absence and enable early return-to-work. The study can increase awareness which in turn can reduce prejudice and stigmatization related to CMDs in working life, because managers’ perspectives are generally considered more objective than employees’ perspectives.

## Conclusions

This study provides managers’ understanding of how CMDs affect capacity to work, adding a new and workplace-based perspective to the increasing knowledge in the field. This perspective is important because managers have an overview of the work context and are in a position to support employees’ capacity to work. Managers’ understanding is that CMDs affect capacity to work with consequences for work output, flexibility and independence, professional appearances, and workplace, customer, and manager interaction. The findings can aid managers to identify employees who struggle at work as well as help managers in how to tailor work accommodations for employees with CMDs, because more in-depth understanding of reduced work capacity is needed to adapt work tasks and workplaces.

## Data Availability

Data supporting the findings of this study are available from the corresponding author upon reasonable request.
